# Transcription termination complex, Rtt103-Rai1-Rat1, regulates sub-telomeric transcripts in *Saccharomyces cerevisiae*

**DOI:** 10.1080/15476286.2023.2192552

**Published:** 2023-03-27

**Authors:** Kathirvel Ramalingam, Krishnaveni Mishra

**Affiliations:** Department of Biochemistry, School of Life Sciences, University of Hyderabad, Hyderabad, India

**Keywords:** Telomeres, telomere position effect, TERRA, Rtt103, Rat1

## Abstract

Telomeres are terminal structures that define the ends of linear chromosomes. They harbour specialized ribonucleoprotein complexes which play a major role in genome integrity by preventing unscheduled DNA damage repair events. Genes located adjacent to telomere repeat sequences are repressed by a phenomenon called telomere position effect (TPE) via epigenetic silencing. RNA surveillance pathways post-transcriptionally regulate any leaky transcripts arising from the telomeres. Recently, multiple non-coding RNA species originate from telomere ends, namely, TERRA (telomeric repeat‐containing RNA), ARRET, sub-telomeric XUTs and sub-telomeric CUTs have been identified. In this study, we report a role for the transcription termination complex (Rtt103-Rai1-Rat1) in regulating the abundance of the sub-telomeric transcripts in a transcription-dependent manner. We show that the Rtt103 mutants have elevated levels of TERRA and other sub-telomeric transcripts that are usually silenced. Our study suggests that Rtt103 potentially recruits the exonuclease, Rat1 in a RNA polymerase II dependent manner to degrade these transcripts and regulate their levels in the cell.

## Introduction

Telomeres, the terminal structure of eukaryotic linear chromosomes, are an array of specialized nucleoprotein complexes. They play a crucial role in maintaining genome integrity by protecting the ends from being recognized as DNA-double strand breaks (DSBs) [1]. Like most organisms, the chromosome ends of *Saccharomyces cerevisiae* consist of a short array of tandem repeats (C_1–3_A/TG_1–3_) that averages approximately 300 ± 75 bp in size [2]. The ends have three well-defined regions, namely, sub-telomeric, middle and repetitive elements, often referred to as TAS elements (Telomere-Associated Sequences). TAS are of two classes, namely, X and Y’. The Y′ elements are either long (6.7 kb) or short (5.2 kb) and are present in 0–4 copies per chromosome end [3]. Y’ is present in only half of the telomeres, whereas X-element is present at all the telomeres. X-element is much more heterogeneous in both sequence and size than Y’ elements. The sub-telomeric regions are highly dynamic and undergo frequent recombination events [4,5]. As a result, they vary significantly in size between closely related strains. Because of the dynamic nature of the TAS elements, the proteins binding to them vary from telomere to telomere and confer distinct functions [6]. The X-elements are generally transcriptionally repressed and devoid of nucleosomes. Y’ elements, on the other hand, are transcriptionally active and contain nucleosomes. Furthermore, Y’ elements do not exhibit the classic characteristics of heterochromatin such as high occupancy of Sir3 and Rap1, and display low levels of histone H4 lysine 16 acetylation [6].

Telomeres are subjected to continuous attrition with each cell division because of the end replication problem [7]. This is counteracted by the action of telomerase, a reverse-transcriptase which utilizes a RNA moiety as a template to lengthen the telomere ends [8]. In the absence of telomerase, the telomeres reach a critically short length after a few cell divisions and undergo an irreversible cell cycle arrest called replicative senescence [9]. In yeast, cells undergoing premature senescence use either amplification of Y’ (Type I), TG_1–3_ repeats (Type II) or TERRA (Telomere Repeat containing RNA) transcripts to lengthen the critically shortened telomeres [10–13].

Another salient feature of the telomeres is the establishment of heterochromatin region. The telomeric tract is non-nucleosomal and has a distinct set of telomere specific proteins [2,14]. The major constituent is the Rap1p which binds DNA in a sequence-specific manner and recruits other proteins, namely, Rif1, Rif2 and SIR complex, which together regulate telomere length and transcription of sub-telomeric genes [15,16]. The adjacent sub-telomere sequence has nucleosomes [14]. Rap1 recruits the Silent information regulator proteins, Sir3 and Sir4 to the telomeres which in turn recruit the NAD-dependent histone acetylase that deacetylates H3 and H4 [16,17]. The association of the Sir proteins and the consequent deacetylation of nucleosomal histones represses transcription in this region [18]. Several additional factors contribute to TPE like chromatin remodellers, chromatin assembly factors, telomere resident proteins (Rif1, Rif2, Yku70/80), telomere folding, insulators and silencing elements at the sub-telomeres and the anchoring of the telomere to the nuclear periphery [18–24]. Silencing initiated from the telomeric tracts is discontinuous and while the X element is effectively silenced in a Sir protein-dependent manner, the Y elements are not [25]. In addition, based on genome-wide transcript analysis, it is clear that only a few genes on a few chromosomes beyond 3.5kb from the telomere tracts are silenced in a Sir protein-dependent manner, while a majority are not. However, the overall levels of transcripts from this region is low and it is not clear if it is due to reduced transcription [26].

Recently, it has become clear that despite the epigenetic silencing of sub-telomeric regions, several RNA species arise from the telomeric and sub-telomeric region. These include TERRA (telomeric repeat‐containing RNA), sub-TERRA, ARRET, ARIA, sub-telomeric XUTs, sub-telomeric CUTs and several other RNA species which originate from telomere ends [27,28]. Some of these RNA species, including TERRA, are possibly universally conserved and have been detected in several organisms including yeasts, plants, parasites and mammals. The current view suggests a strong role for TERRA to be a part of the telomeric architecture as they remain physically associated with the telomeres [29,30]. Expression of TERRA is cell cycle regulated and ideal levels of TERRA are crucial for the cellular fitness as either overexpression or downregulation causes telomere dysfunction-induced foci [12,29,31–35].

In *S. cerevisiae*, TERRA is an RNA Pol II product which is regulated both epigenetically [36] and post-transcriptionally via RNA surveillance pathways [27,30]. In X-only containing telomeres, the repression of TERRA is mediated by both Sir2/3/4 and Rif1/Rif2-Rap1 complex. On the other hand in Y’ containing telomeres, the repression is mediated by primarily Rap1- Rif complex [36]. The turnover of TERRA, irrespective of its origin, is mainly mediated by Rat1, a nuclear 5’-3’ exonuclease [30]. But the exact molecular mechanism of TERRA turnover still remains elusive.

The transcription cycle of RNA Pol II is a highly coordinated event which is regulated via its C-Terminal Domain (CTD) [37]. In yeast, the CTD comprises 23 consecutive hexapeptide repeats (Y_1_-S_2_-P_3_-T_4_-S_5_-P_6_-S_7_) that are dynamically phosphorylated and dephosphorylated [38]. At the promoter, the CTD mostly remains unphosphorylated [39]. As it transverses through the gene body, Ser5 phosphorylation (Ser5P) dominates and aids in the recruitment and activation of 5′- capping enzymes [40,41]. Ser5P gradually decreases as RNA Pol II progresses towards the elongation phase and phosphorylation of Ser2 (Ser2P) increases [39]. Towards the 3’end, Ser2P dominates and is involved in the recruitment of polyadenylation, cleavage and termination factors [42]. Rtt103p is one such transcription termination factor which interacts with the Ser2P RNA Pol II via its CTD interacting Domain (CID) and is thought to cooperatively recruit Rai1 and Rat1 to the 3’ end of the gene body, promoting termination of transcription [43]. Apart from termination of transcription of mRNA, Rat1 and Rai1 are involved in processing the pre-rRNA transcripts [44–47].

A genome-wide distribution analysis of RNA Pol II revealed its enrichment in various ‘epigenetically silenced’ regions of the genome like the telomeres, rDNA ‘E-Pro’ region, centromeres, HML and HMR loci. Interestingly, it was shown that the transcriptional regulation at the rDNA is via the alternative transcription termination pathway Nrd1-Nab3-Sen1 [48,49]. The non-coding RNA (IGS1-R) arising from the rDNA is proposed to recruit Nrd1-Nab3-Sen1, which in turn regulates the levels of these transcripts in conjunction with TRAMP and exosome complex [49]. Furthermore a single amino acid substitution in Sen1 (E1597K) resulted in aberrant synthesis of many regulatory noncoding RNAs and mRNAs [48]. These studies suggest that there might be a fine tuning of transcript levels by further action of the transcription termination factors.

In our study, we report a novel role of transcription termination complex Rtt103, Rai1 and Rat1 in sub-telomeric gene silencing and in the regulation of the long non-coding RNAs arising from the sub-telomeric and telomeric tracts. Further, we demonstrate that this complex is physically enriched at the telomeres. The recruitment of the Rat1 exonuclease to the terminus is dependent on Rtt103 and requires active transcription suggesting a potential mechanism for the enrichment of this complex at the telomeres.

## Results

### rtt103Δ mutants are defective in sub-telomeric silencing

**Figure 1.**
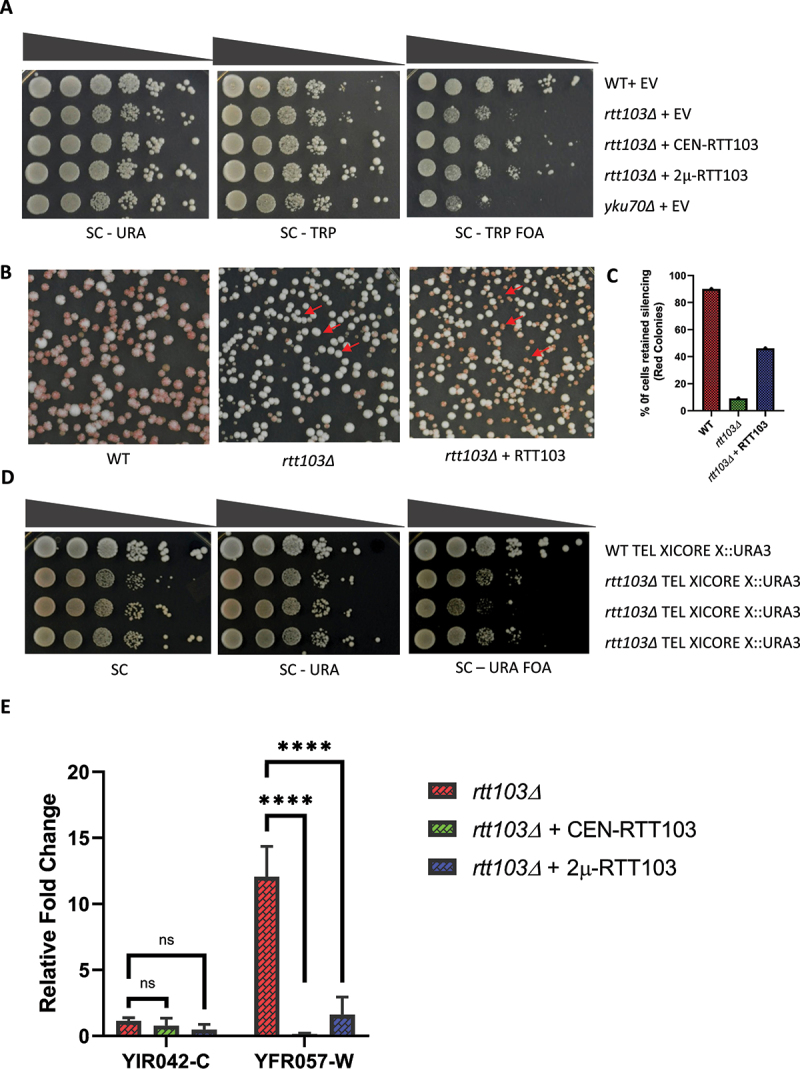
*rtt103Δ* is defective in sub-telomeric silencing.

Note: A. Indicated strains transformed with either empty vector (EV) or full length Rtt103 encoded on a single copy CEN vector (CEN) or multicopy 2 micron vector (2 μ) were grown overnight and 5-μl of tenfold serial dilutions were spotted on SC-TRP, SC-TRP URA and SC- TRP+FOA plates. Plates were imaged after 72 hours of growth at 30°C.

Previous investigation in *S. cerevisiae* to identify suppressors for *yku70*Δ temperature-sensitive phenotype yielded high copy expression of *RTT103* as a partial suppressor for temperature sensitivity [50]. Although we found *RTT103* does not have a role in repairing non-chromosomal substrates via non-homologous end joining (data not shown), a key function of Yku70, we extended the study to test whether Rtt103 has any role in telomere metabolism and gene silencing, another important function of Yku70. Transcription of genes located adjacent to telomeres is usually repressed by the telomere position effect (TPE) [19]. To assess the impact of Rtt103 in sub-telomeric silencing, we generated *rtt103Δ* in a strain that harbours *URA3* at the telomeric region of chromosome VIIL. The expression of *URA3* in this locus is silenced by TPE [51]. The measurement of loss of silencing was performed by spotting overnight grown cultures on the agar plates containing 5- fluoroorotic acid (5-FOA) in growth medium. The *URA3* gene encodes for an enzyme orotidine-5’-phosphate decarboxylase, which converts the 5-FOA into a toxic substance 5’ fluorouridine monophosphate. Hence, loss in silencing restricts the growth of the cell in 5-FOA, and conversely, robust growth on plates containing 5-FOA indicates silencing. Wild-type cells exhibit TPE. As silencing at the telomeres is stochastic, wild-type cells grow on both SC-URA and SC + 5-FOA plates. But in an *rtt103Δ*, we found reduced silencing, as indicated by the reduction in growth on 5-FOA plate, compared to wild type (Figure 1A). *yku70Δ*, which is known to have strong silencing defects, was used a positive control and is severely defective for growth on 5-FOA plates. Silencing was restored upon complementation with either single copy (CEN) or multicopy (2 micron) *RTT103* (Figure 1A).

B. Wild type, *rtt103Δ* and *rtt103Δ* carrying full length Rtt103 in CEN vector were grown overnight in synthetic complete media, serially diluted tenfold and 100 μl was plated on YPD. Plates were imaged after 72 hours of growth at 30°C.

C. Bar graphs depicting the percentage of cells that retained silencing (red and red sectored colonies) has been quantified (n = 500).

D. WT and *rtt103Δ* harbouring TEL XICORE X:URA3 were grown overnight in synthetic complete media, serially diluted tenfold and 5 µl was spotted on SC, SC-URA and SC-FOA. Plates were imaged after 72 hours of growth at 30°C.

E. RNA was isolated from the indicated strains and converted to cDNA. cDNA was then subjected to qRT-PCR to assess the relative expression of YIR042-C and YFR057-W. All data are depicted as mean + SEM, n = 3. P values were obtained from two-way ANOVA.

Further, we used a colour-based assay to study the loss of silencing by integrating the *ADE2* marker at the telomeric region of chromosome VIIL. *ade2Δ* cells display a red colony colour phenotype on rich yeast growth medium, YPD. The red colour is due to the accumulation of intermediate metabolites of the adenine biosynthesis pathway. When *ADE2* is inserted proximal to the telomere sequences, wild type cells exhibit TPE and do not continuously express *ADE2*. So they appear red like an *ade2Δ* mutant, stochastically switching to white for a few generations, leading to the appearance colonies with red and white sectors (Figure 1b first plate). In an *rtt103Δ*, due to loss of silencing, 90% of the cells appeared white with no red sectors (Figure 1B,C). We noticed that in this case that the complementation with plasmid encoded Rtt103 is partial. We also generated a *rtt103Δrai1Δ* double mutant in the telomere VIIL ADE2 background and found that the silencing defect was even more severe and almost no red sectors were seen (Figure S1A). However, this strain was extremely sick and was not used for further studies.

Several studies suggest discrepancies in the silencing levels between natural telomeres and modified truncated telomeres [25]. TPE was initially studied by placing either *URA3* or *ADE2* gene immediately adjacent to the telomeric TG_1–3_ tract by removing the adjacent X and Y′ elements [52]. Moreover, TPE varies substantially from telomere to telomere in the same cell and between different strain backgrounds. It is mainly due to the difference in sub-telomeric structures at each telomere and the differential occupancy of silencing proteins in X and Y’ elements [23,36,53]. Although Y’ elements have high nucleosome density, they are transcriptionally active. They lack the classical hallmarks of heterochromatin, such as high Sir3 and Rap1 occupancy as well as low levels of histone H4 lysine 16 acetylation [6,18,54]. Therefore, to confirm whether the silencing defect observed in *rtt103Δ* is not specific to modified truncated telomeres, we also assessed the silencing defect of *URA3* marker inserted at the core X of chromosome XI-L of unmodified natural telomeres [55]. As shown in (Figure 1D), *rtt103Δ* strain has lowered growth in 5-FOA while growing robustly on SC-URA plates, indicating reduced silencing at the native telomeres as well. In order to further validate the silencing defect in *rtt103Δ*, we also assessed the relative levels of two native sub-telomeric genes namely *YIR042-C* and *YFR057-W* via quantitative reverse transcriptase PCR (qRT-PCR) [56]. *YIR042-C* is located at chromosome IX and 3.9Kb away from the telomere end, whereas *YFR057-W* is located at chromosome VI at 645bp away from the end and have been shown to be silenced in a Sir-protein-dependent manner [26]. qRT-PCR results revealed that in an *rtt103Δ*, there was a 12-fold increase in the relative amounts of *YFR057-W* in comparison with WT. We could only detect a modest increase in YIR042-C transcripts that was not statistically significant (Figure 1E). Complementation with either a single copy (CEN) or multicopy (2μ) *RTT103* restored silencing completely. Taken together, these data establish that silencing at the sub-telomeres is compromised in *rtt103Δ* compared to wild type.

### *rtt103Δ*, *rai1Δ* and *rat1–1* are defective in sub-telomeric silencing

**Figure 2.**
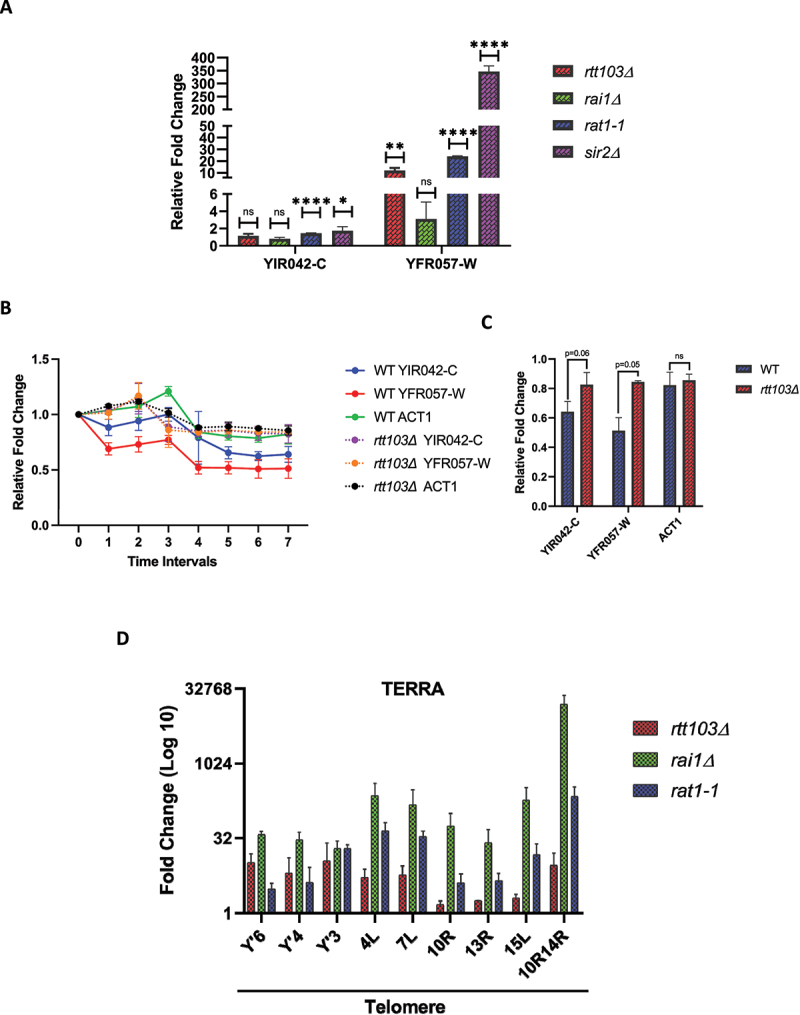
*Rtt103Δ, rai1Δ*, and *rat1–1* are defective in sub-telomeric silencing.

Note: A. Expression of YIR042-C and YFR057-W was assessed by qRT-PCR in the indicated strains. Fold change with respect to wild type is shown. All data are depicted as mean + SEM, n = 3. P values were obtained from student’s t-tests.

As Rtt103 is proposed to work in concordance with Rat1 and Rai1 in transcription termination, we decided to test the role of this complex in telomere silencing. We assessed the level of silencing defect in *rai1Δ* and *rat1–1* at the two native sub-telomeric genes, namely, YIR042-C and YFR057-W, via quantitative reverse-transcriptase PCR (qRT-PCR). Rat1 is an essential gene and the *rat1–1* allele is temperature sensitive. Therefore, the silencing was assessed at 39°C in *rat1–1*. All three mutants revealed a varying range of silencing defects for YFR057-W in comparison with wild type (Figure 2A) with *rai1Δ* showing moderate increase in transcript abundance. *SIR2*, which is directly involved in epigenetic silencing of sub-telomeric loci, was used as a positive control. Again, YIR042 transcript levels were increased slightly in *rtt103Δ* and *rai1Δ* but showed a more pronounced increase in *rat1–1* and *sir2Δ*. As loss of all three proteins leads to increased accumulation of telomeric and sub-telomeric transcripts, it is possible that Rat1, Rai1 and Rtt103 together have a role at the telomeres as in transcription termination.

B. *rtt103Δ* alters the stability of sub-telomeric transcripts. qRT-PCR analysis of the indicated sub-telomeric transcripts in *rpb1-1* and *rtt103Δ rpb1-1* after shifting to non-permissive temperature. RNA at the time 0-time point was arbitrarily set to 1. ACT1 was used as a control. The data from two independent experiments were plotted, and the error bars represent the standard deviation at each time point.

C. Relative abundance of YIR042-C, YFR057-W and ACT1 transcripts for the last time point was assessed in the indicated strains. All data are depicted as mean. P values were obtained from Student t-test (un-Paired).

D. TERRA accumulates in *rtt103Δ, rai1Δ and rat1-1*. RNA was isolated from the indicated strains and qRT-PCR with TERRA specific primers was performed to assess the levels in Y′ and X‐only containing telomeres. Three independent experiments were carried out, and an average of the three with standard error is plotted above. The primers Y’6/Y’4/Y’3/10R14R detect TERRA transcripts from more than one chromosomal end.

### *rtt103Δ* alters the stability of sub-telomeric transcripts

From the existing literature, it is evident that sub-telomeric transcripts are regulated epigenetically [19], transcriptionally [30] and post-transcriptionally via RNA surveillance pathways [27,57]. In order to address whether it is exacerbated transcription rate or stability of the sub-telomeric transcripts that is altered in an *rtt103Δ*, we employed a temperature-sensitive allele of the RNA Pol II subunit, *rpb1–1*. The transcription can be turned off by inactivating RNA Pol II after shifting from permissive (25°C) to non-permissive temperature (39°C), whereas RNA Pol I and Pol III transcription status remains unaltered. We analysed the stability of transcripts arising from *YFR057-W* and *YIR042-C* along with *ACT1* at regular intervals after inhibiting transcription. Total RNA was isolated from samples harvested at 0 minutes (just before the temperature shift) and every half an hour after the temperature shift to 39°C. Relative abundance of sub-telomeric transcripts and *ACT1* transcripts was obtained by normalization to 7S RNA and plotted as a function of time. 7S RNA is a non-coding RNA arising from the *SCR1* locus, is transcribed by RNA polymerase III, and is unlikely to be affected by defective RNA polymerase II function at higher temperatures. In comparison with wild type (*rpb1–1*), *rtt103Δ rpb1–1* revealed greater stability of sub-telomeric transcripts, whereas stability of *ACT1* transcript remained the same in both (Figure2B and 2C). These data suggest that in an *rtt103Δ* mutant, it is the stability of the transcript, not the rate of transcription, which is altered and it is also specific to sub-telomeric transcripts.

### TERRA accumulates in an rtt103Δ, rai1Δ and rat1–1

Recent discoveries of regulatory ncRNAs controlling cis and trans gene silencing in budding yeast have led to speculation that these RNAs might be directly involved in heterochromatin regulation at the telomeric regions [58–61]. In the RNA-Seq analysis of a *rtt103Δ* strain (Kathirvel and Mishra unpublished), we found several of the putative Y’ helicases encoded within Y’-element of the sub-telomere region were up-regulated by more than twofold. In *S. cerevisiae* there are 11 Y′-Long elements and eight Y′-Short elements [11]. Each encodes more than one open reading frame (ORF) which potentially confers helicase activity [11,62]. In telomerase negative cells, these transcripts act as a template to alternatively lengthen the chromosome ends (ALT-Type I survivors) [10,11]. We first tested if the Y’helicases were indeed upregulated in the *rtt103Δ* strains via RT-PCR using single primer set to measure highly conserved Y’ helicases from seven distinct loci. We find that Y’ helicases are upregulated in *rtt103Δ* and *rai1Δ* (Fig S1B).

In addition to ORFs for putative helicases, the sub-telomeric region also contains promoter like elements for TERRA [28,63]. The TERRA sequence overlaps with the Y’ helicases and ranges from ∼100–1200 bases in size. TERRA is a major class of non-coding RNA that is produced from the sub-telomeric region and is conserved from yeast to humans [64]. In yeast, TERRA is an RNA Pol II product and has a 3’ poly-A tail [27,30]. Its expression is tightly regulated via a Rap1p and Rat1p dependent mechanism and RNA surveillance pathways [12,27,30]. TERRA mostly remains associated at the telomeres suggesting a potential regulatory role in telomere replication and architecture [64,65] although precise functions of the TERRA transcripts still remains elusive. Therefore, we assessed the levels of TERRA from multiple chromosomes in an *rtt103Δ, rai1Δ* and *rat1–1* as described by Iglesias et al., 2011 via quantitative real-time PCR protocol [36] (Figure 2D, Fig S1C). We found that TERRA levels were increased in all the three mutants and *rai1Δ* had a larger level of accumulation compared to both *rtt103Δ* and *rat1-1*. While it is known that Rat1 plays a key role in keeping TERRA levels low, we find that both its partners, Rtt103 and Rai1, also contribute to regulating TERRA levels. These results suggest that, as proposed for transcription termination, the Rat1-Rai1-Rtt103 could work together in the regulation of TERRA.

### Rtt103p recruits Rat1p to telomeres in a transcription dependent manner

**Figure 3.**
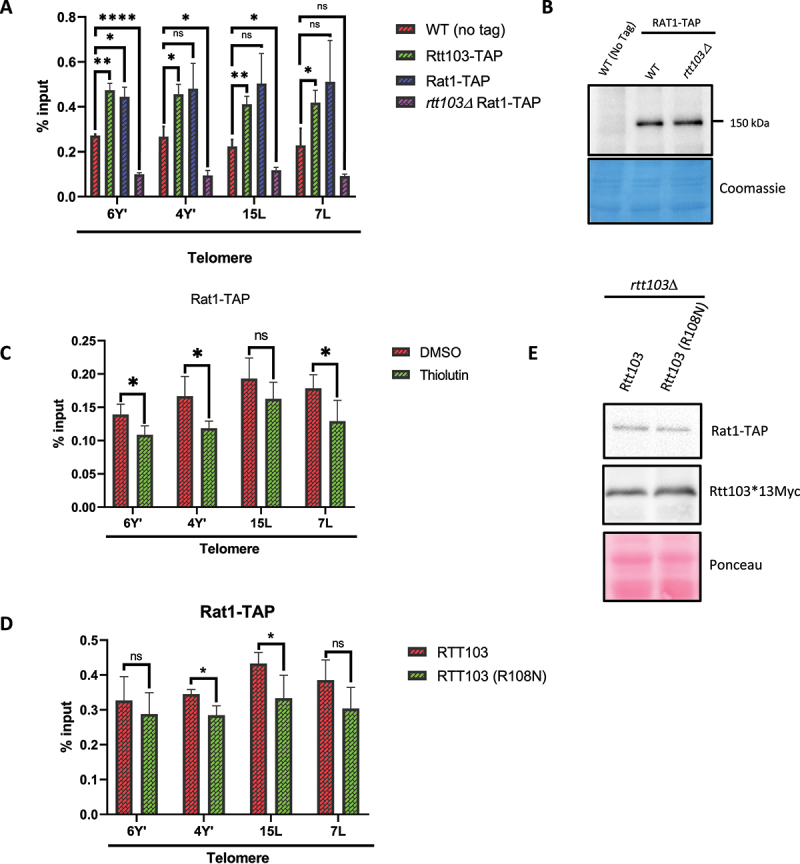
Rtt103p is physically associated with the telomeres.

Note: A. ChIP using IgG sepharose was performed with the indicated strains encoding TAP tags. Enrichment of Rtt103 and Rat1 at the indicated telomeres was analyzed. The WT without tag was used as a background control. The experiments were carried out three times, and the error bar represents the standard deviation. Statistical significance was calculated using two-way ANOVA (*p < 0.05).

It has been reported that Rat1p associates with the telomeres during late S-phase when the telomere gets replicated and this association is dependent on the continued presence of Rif1 and Rif2 [12]. The association is abolished upon telomere shortening [12]. Moreover, recent work to identify telomere‐associated proteins in *S. cerevisiae* via telomere-mimetic sequence as bait revealed both Rat1 and Rai1 are physically associated at telomeres [66]. In transcription termination, Rtt103 is proposed to be a recruiter for Rai1 and Rat1 by virtue of its interaction with RNA Pol II [43,67]. We, therefore, tested whether Rtt103 acts as a recruiter of Rat1 to the telomeres as well. We generated *rtt103Δ* in strains encoding TAP-tagged Rat1. TAP-ChIP was performed in Rtt103-TAP, Rat1-TAP and *rtt103Δ* Rat1-TAP strains and association with a few specific Y elements was measured. First, we found that Rtt103 is enriched at the telomeres to similar extents as that of Rat1. Second, in the absence of Rtt103, much lower levels of Rat1 could be detected (Figure 3A). As the Rat1 protein levels remain unaltered in an *rtt103Δ* (Figure 3B), it is the recruitment of Rat1 to the telomeres that is impaired significantly.

B. Western blot analysis of expression of Rat1-TAP in WT and *rtt103Δ*. Rat1 protein levels remain comparable to WT in *rtt103Δ*. Blot was stained with Coomassie after developing as a loading control.

C. ChIP was performed for Rat1-TAP in the presence and absence of thiolutin. Enrichment at the TERRA/Y’ loci was measured via qPCR and mean values were compared to their respective input. The experiment was done in triplicates and the mean was plotted. The error bars represent the standard deviation of mean and statistical significance was obtained using two tailed Student t-tests (*p < 0.05).

D. ChIP was performed for Rat1-TAP in an *rtt103Δ* harbouring ectopically expressed WT *RTT103* or the point mutant *RTT103 (R108N)*. Enrichment was measured via qPCR and mean values were compared to their respective input. All data are depicted as mean + SEM, n = 3. P values were obtained from two-tailed Student t-test (*p < 0.05).

E. Western blot analysis to assess the expression of Rat1-TAP in *rtt103Δ* harbouring Rtt103 (R108N). Ponceau stained blot serves as a loading control.

To further delineate whether this recruitment is transcription-dependent, we performed ChIP for Rat1 in the presence of thiolutin – a well-known inhibitor of yeast RNA polymerases [68]. Upon inhibition of transcription, there was substantial reduction in the enrichment of Rat1p at the most Y’ regions tested (Figure 3C). We also performed ChIP with an already known point mutant of *RTT103* (R108N) which has reduced interaction with RNA Pol II as determined by anisotropy and NMR measurements [69]. The enrichment of Rat1-TAP at the telomeres was reduced in the strains harbouring *RTT103* (R108N) in comparison with the strain with wild type *RTT103* (Figure 3D) without any reduction in the total amount of Rat1-TAP protein in both strains. (Figure 3E). This suggests that it is the recruitment of Rat1p to telomeres is affected and strengthens the idea that atleast some Rat1 is recruited to telomeres via Rtt103 in a transcription-dependent manner.

## Discussion

In this work, we demonstrate that Rtt103, a transcription termination factor, is required for efficient silencing of telomeric and sub-telomeric transcripts. The telomere length of *rtt103Δ* remains unaltered (Figure S1D). Similar to their role in transcription termination, both Rtt103 partners, Rai1 and Rat1, are also involved in regulating the levels of these transcripts. Of note, we show that Rtt103 could recruit Rat1, the exonuclease that that has been implicated in the degradation of these transcripts. We show that association of Rtt103 with the RNA polymerase II is required for regulating the levels of transcripts and that this association is likely mediated via interaction of the C terminal domain of RNA polymerase with Rtt103.

Several recent high-throughput transcriptional analyses have now established that transcription of telomeres and sub-telomeres is a conserved phenomenon among different phyla [27,29,32,63,70,71]. In humans, the TERRA length varies from few hundred to around 9Kb in size and is transcribed in a centromere to telomere orientation and most of the population is 7-methylguanosine (m7G) capped at the 5’ ends while only 7% is polyadenylated [29]. In yeast, TERRA is transcribed from both Y’ and X-only containing telomeres [36]. Their average size ranges from ∼100–1200 bases and all are polyadenylated, while 5’ m7G cap has not been demonstrated directly [72]. The TERRA transcripts mostly originate in the sub-telomeres suggesting a defined transcription start site. In humans, CpG islands on a sub-set of telomeres appear to be promoters and cytosine methylation at these sites negatively regulates transcription [28,63]. Since there appears to be defined initiation sites, the heterogeneity of TERRA size might be due to differential termination or processing of 3’ ends. TERRA molecules lack the conserved poly-adenylation and cleavage signal 5′-AAUAAA-3′; and the mechanism of transcription termination remains elusive. In general, protein coding genes that contain the polyadenylation signal are terminated by the Rtt103-Rai1-Rat1 complex. However, for TERRA, earlier studies and our work suggest that the Rtt103-Rai1-Rat1 complex regulates stability and we speculate that termination mechanism may also be similar to that of protein coding genes.

In an *rtt103Δ*, silencing at the sub-telomeres is compromised at both modified and natural telomeres (Figure 1A–). All the three transcription termination mutants revealed varying degrees of silencing defects for the sub-telomeric genes, Y’ helicases and TERRA (Figure 2). Increasing evidence implying that Y’ helicases and TERRA transcripts are being used as templates in telomerase negative cells makes us speculate this termination complex may have an important role to play in regulating telomere length in the absence of telomerase [11,12]. Although they work together in maintaining the levels of sub-telomeric transcripts, the variation in silencing might be due to impaired recruitment of Rat1 to the telomeres in case of *rtt103Δ* (Figure 3a), whereas in an *rai1Δ* mutant, it might be the compromised 5’-3’ exonuclease activity exhibited by Rat1, as it has been reported that Rai1 enhances Rat1 activity in vitro [44]. In addition, Rai1 possesses a decapping, pyrophosphorylase and exonuclease activity as well and recognizes unmethylated Gppp caps [73–76]. We speculate that some of the transcripts may be degraded by the activity of Rai1 in a co-transcriptional manner before the RNA is fully capped and protected. This could also be why *rai1Δ* have increased TERRA compared to both *rtt103Δ and rat1–1*. Also decapping by Rai1 would expose a 5’phosphate that would make TERRA a substrate for Rat1.

In an *rtt103Δ*, the stability of the sub-telomeric transcripts is specifically altered, implying that these transcripts might be co-transcriptionally regulated in a Rat1-dependent manner (Figure 2b,c). Here we report that the enrichment of Rat1 to the telomeres is via Rtt103 in a transcription-dependent manner (Figure 3c). Previous studies have reported that when a telomere gets shortened, the association of Rat1 to the telomere is abolished and the continued presence of Rif1 and Rif2 is required for the association [12]. The absence of Rat1 at the telomeres in *rif1* and *rif2* could be due to the increased TPE and hence inaccessibility to the transcription machinery [77,78]. Alternately, Rat1 could be recruited independently by both Rap1/Rif1/Rif2 and Rtt103 to telomeres. It is known that TERRA exists in three different fractions, namely, chromatin associated, nucleoplasmic and cytoplasmic fraction [79]. It has been suggested that the non-chromatin associated fraction is regulated via degradation by Rat1 [12]. Here in this study, we report the regulation of TERRA levels by the Rtt103-Rai1-Rat1 termination complex and propose a mechanism by which Rat1 may be targeted to the TERRA RNA.

How might Rtt103 regulate sub-telomeric transcript levels? We envision multiple possibilities (Figure 4a and b). At the X elements where Sir-dependent silencing is robust, we propose that it is the escape transcription that is regulated by this complex as proposed earlier [80]. Normally, Sirp-dependent epigenetic silencing silences much of the transcription; any transcription that is initiated is prematurely terminated and if transcription is completed then it is subjected to exosomal degradation. We suggest the improper termination of transcription at the inserted *URA3* locus leads to production of transcripts with longer 3’ ends that might be poor substrates for exosomes, leading to export of this transcript and translation. At the non-coding TERRA site, one possibility is the co-transcriptional recruitment of Rat1 (via Rtt103) to the sub-telomeric transcripts leads to degradation. As Rat1 can only act on uncapped 5’ ends, we think that once the RNA is capped, it has to be decapped and then Rat1 can degrade. This is possibly post-transcriptional. Alternately or additionally, Rai1 could also independently exhibit this activity on nascent transcripts co-transcriptionally once recruited to the transcribing polymerase as it possesses a decapping and exonuclease activity on unmethylated 5’ caps [73–76]. As loss of Trf4, which is involved in targeting RNA to nuclear exosome, also increases TERRA abundance (albeit a minor one), it is possible atleast some of the TERRA is targeted to the nuclear exosome. We suggest that the TERRA transcripts produced in the absence of Rtt103-Rai1-Rat1 may have abnormal 3’ ends and may not be degraded by the exosome machinery efficiently leading to increased accumulation of these transcripts [81]. In sum, we propose that free TERRA is kept at very low levels in wild type by the combined action of Rtt103, Rai1 and Rat1 by targeting it in a co-transcriptional manner.

**Figure 4a.**
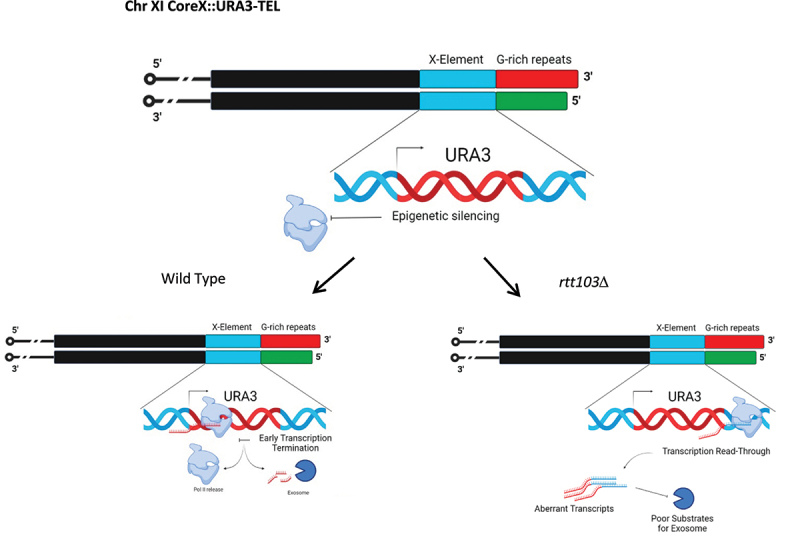
**Silencing at X-element heterochromatic loci:** In a wild type heterochromatic loci are kept repressed via three independent mechanisms. 1. Epigenetic silencing – Heterochromatin formation and repression of RNA Pol II via SIR complex (major pathway). 2. Premature termination of transcription 3. Any leaky mature transcripts will be targeted for exosome mediated degradation. In an *rtt103Δ* we speculate that there could be transcription read-through due to impaired recruitment of Rai1 and Rat1 for proper termination. As such aberrant transcripts are poor substrates for exosome mediated degradation they exhibit increased stability in *rtt103Δ*.

**Figure 4 b.**
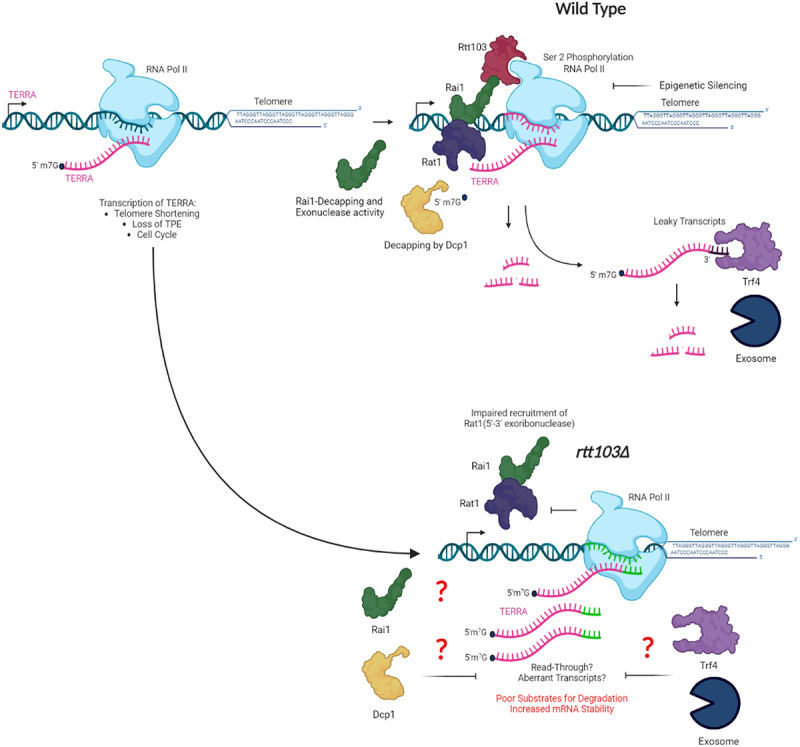
**Co-Transcriptional regulation of TERRA** : In a wild type, the TERRA levels are kept repressed via Rap1 through Rif1/2 and Sir2/3/4 complex and by Rat1 exonuclease activity. A minor pathway that regulates TERRA is the post-transcriptional degradation via TRAMP-mediated exosome targeting. In our study, we speculate there may be co-transcriptional regulation of TERRA by the Rtt103-Rai1-Rat1 complex. Rtt103 recruits Rai1 and Rat1 in a RNA polymerase-dependent manner to the TERRA locus a) where Rai1 exhibits its pyrophosphohydrolyase activity towards mRNA lacking 5’-end cap and perhaps prepares the substrate for Rat1 mediated exonuclease activity. Absence of Rtt103 leads to impaired recruitment of Rai1 and Rat1 resulting in reduced Rat1 mediated degradation of TERRA. It also potentially leads to read-through transcripts which are poor substrates for exosome-mediated degradation.

Interestingly, Rtt103 was initially isolated in a screen for mutants that elevated Ty1 transcription and it was demonstrated that there was a moderate increase in Ty1 transcripts in *rtt103Δ* [82]. This raises the intriguing possibility that Rtt103 could be involved in negatively regulating Ty1 transcripts and in its absence, Ty1 transcripts are stabilized leading to increased cDNA and increased transposition. In another possible link, Y’ helicase transcripts are also higher in *rtt103Δ* and it is known that Y’ helicase is incorporated into the viral like particles produced in the Ty1 transposition cycle [10]. Together these observations suggest a potential role for RTT103-mediated RNA stability in processes affecting transposition and hence genome stability.

There are a number of key questions that remain to be addressed. Induction or overexpression of TERRA from a single telomere induces early-onset senescence [83]. Does stabilization of TERRA also have similar effects? Is transcription of TERRA a coordinated event from all the telomeres? In the case of sub-TERRA and ARIA, it is transcribed from telomere end towards the centromere. Do the telomere ends also possess promoter like elements? In *S. cerevisiae,* sub-TERRA XUT and sub-TERRA CUT are complementary to each other and can form dsRNA. As RNAi does not exist in *S. cerevisiae*, could RNA degradation pathways be more critical to regulate the levels of such non-coding telomere transcripts?

The sub-telomeric regions reveal higher levels of recombination leading to faster evolution of gene families residing in this locus [84–86]. This allows faster and better adaptive responses to the changing environment. Additionally, it contributes to antigenic variation and virulence in some pathogenic yeasts and parasites [28,87–89]. Therefore, understanding regulation of telomeric and sub-telomeric transcription has implications beyond yeast. While this study shows which transcription termination complex involved in such regulation, a study of biological cues which decide between maturation or co-transcriptional degradation might provide effective ways to inhibit transcription of genes which are sub-telomeric in origin. The sub-telomeric region also harbours various other classes of genes like *tlh* which is regulated under nitrogen starvation, cri-TER a temperature-dependent non-coding RNA, genes responsible for biofilm formation [90–92]. It remains to be investigated if expression of these genes are also regulated post-transcriptionally.

## List of strains and plasmids used in this study

The *Saccharomyces cerevisiae* strains used in this study are derivatives of W303 or BY4741 and are listed in Table 1. Strains were grown under standard conditions in YPD (Yeast Peptone Dextrose) or synthetic complete (SC) medium at 28°C. For temperature-sensitive (*ts*) mutants, further specifications are mentioned in the methods section. Standard procedures were followed for yeast manipulations. Either micromanipulation or PCR-based homology-dependent transformation was employed to generate knockouts [93].

**Table:** 

Strain No.	Genotype	Source
KRY 105	W303 *adh4::ADE2* Tel VII L MAT-a	Lab collection [50]
KRY 193	W303 *adh4::URA3* Tel VII L MAT-a	Lab collection [50]
KRY171	yku70:KanMx adh4:Ade2 rad5+mat a	Lab collection [50]
KRY 230	KRY 105 except *rtt103Δ*::KanMx MAT-α	Lab collection [50]
KRY 285	KRY 193 except *rtt103Δ*::KanMx MAT-α	Lab collection [50]
KRY 172	KRY 193 except *yku70Δ*::KanMx MAT-α	Lab collection [50]
KRY 632	*rai1Δ*::KanMx *his3Δ1 leu2Δ0 ura3Δ0* MAT-α	Arlen Johnson
KRY 634	*rat1–1*^*ts*^*ura3–52 leu2Δ1 his3Δ200 trp1Δ63* MAT-α	Arlen Johnson
KRY 2187	yRP693 *ura3–52 leu2 rpb1–1*^*ts*^	Carolyn Decker
KRY 2188	KRY 2187 except *rtt103Δ*::KanMx	This Study
KRY 2189	BY4741 RTT103-TAP:HIS3Mx6	Horizon Discovery
KRY 2190	BY4741 RAT1-TAP:HIS3Mx6	Horizon Discovery
KRY 2191	KRY 2190 except *rtt103Δ*::KanMx MAT-α	This Study
KRY2234	KRY105 except *rtt103Δ*::KanMx *rai1Δ*::KanMx MAT-α	
KRY 931	YEF505 ura3, leu2, ade2Δ, telXI coreX:URA3, SIR3-GFP:TRP1 MATα	Emmanuelle Fabre [55]
KRY 2192	KRY931 except *rtt103Δ*::KanMx	This Study
CKM 261	Full length RTT103 in YCplac22	Lab collection [50]
CKM 285	Full length RTT103 in YEplac112	Lab collection [50]
CKM 287	RTT103*13xMyc in pBEVY-T	This study
CKM 767	RTT103 (R108N) *13Myc in pBEVY-T	This Study

## Methods

### Yeast spot growth assay

For spotting assays, yeast cells were grown overnight at 28°C in appropriate selection media. Cells were harvested at OD_600_ − 1 and ten-fold serial dilutions were made and 5 µl spotted onto appropriate agar plates. Plates were then incubated for 2–3 days at 28°C and photographed. The FOA concentration used is 1 mg/ml. For the *ADE2* colour-based silencing assay, cells were directly plated on YPD agar plates, incubated at 30°C for 2 to 3 days and photographed.

### RNA preparation and c-DNA synthesis

Total RNA was isolated by extraction with hot acidic-phenol (pH-5) as described by Collart and Oliviero [94]. For RNA half-life experiments, cells were grown in SC medium- to mid-log phase at permissive temperature (25°C), centrifuged and shifted rapidly to non-permissive temperature (39°C) by adding equal amount of pre-warmed medium (50°C) and incubated at 39°C for the respective intervals. For *rat1–1 ts* mutant, the cells were sub-cultured and grown up to OD_600_ 0.5 ~ 0.8 (28°C) and shifted to non-permissive temperature (39°C) for 3 hours. For data normalization of *rat1–1*, similarly treated wild-type cells were used. For DNaseI digestion, 3 μg of RNA was subjected to digestion with 10 units of DNaseI (New England Biolabs) enzyme at 37°C for 3.5 hours to achieve an RNA which is completely devoid of telomeric DNA. Before c-DNA synthesis, a normal PCR (40 Cycles) was performed with primers specific for telomere and *ACT1* to assess the DNA contamination. RNA alone was also employed as a template for qRT-PCR to confirm the purity of the samples. The reverse transcription was performed using Verso cDNA Synthesis Kit (Thermo Scientific) as per the manufacturer’s protocol at 55°C for 60 min followed by inactivation at 95°C for 2 min.

### TERRA level analysis by qRT-PCR

TERRA reverse transcription was done using 10 μM CA oligonucleotide and 2 μM *ACT1* oligonucleotide in a final volume of 20 μl. For qPCR, the cDNA was diluted with equal volume of nuclease-free H_2_O. A volume of 1 μl cDNA was quantified in a final volume of 10 μl reaction by real-time PCR with the Power SYBR Green PCR Master mix (Applied Biosystems) using an Quanstudio3 Real-Time PCR System. The final concentration for each primer set differs from 0.2 to 0.6 μM as described by Iglesias *et al*., 2011 [36]. The reactions were incubated for 10 min at 95°C, followed by 40 cycles of 15 s at 95°C and 1 min at 59°C. TERRA levels were normalized to respective actin values and compared to the isogenic wild type. The primers Y’6/Y’4/Y’3/10R14R detects TERRA stemming from more than one chromosomal end (6Y’- 8 L/8 R/12 L-YP1^a^ /12 R-YP2^a^ /13 L/15 R) (4Y’-9 L/10 R/12 R-YP2^a^ /15 R) (Y’3-12 L-YP1^a^ /12 R-YP2 ^a^ /15 R). Whereas for X-only containing telomeres, individual primers have been designed − 4 L, 7 L, 10 R, 13 R, 15 L, 10R14R.

#### Chromatin immunoprecipitation

ChIP was performed as described [95]. Briefly, yeast cells were grown to OD_600_ 0.8–1 and crosslinked for 10 mins with formaldehyde (final conc. 1.2%) and quenched with glycine (360 mM) for 15 mins. Cells were pelleted and washed twice with 1× TBS, resuspended in lysis buffer (0.1% deoxycholic acid, 1 mM EDTA, 50 mM HEPES/KOH pH-7.5, 140 mM NaCl, 1% Triton X-100, protease inhibitor cocktail) and lysed using 0.5-mm glass beads in a vortex mixer for 20 min at 4°C. The chromatin lysate was recovered and sheared 10 sec on/off (Henderson Biomedical MSE/Amplitude 10) for 5 cycles. An input sample representing 10% of the ChIP extract was employed as input for normalizing the qPCR. 80 μl bed volume of IgG Sepharose beads were washed with lysis buffer and IP was performed overnight at 4°C. Beads were washed with lysis buffer P500 (0.1% Deoxycholic acid, 1 mM EDTA, 50 mM HEPES/KOH pH7.5, 500 mM NaCl, 1% Triton X-100), + LiCl detergent buffer (0.5% deoxycholic acid, 1 mM EDTA, 250 mM LiCl, 0.5% NP-50, 10 mM Tris-Cl pH8) + 2× TBS. The bead-bound DNA was eluted in 100 μl (1% SDS/1× TBS) + 150 μl (1% SDS/1× TBS) for 10 min at 65°C. For reversing the crosslink, input and IPs were treated overnight at 65°C with proteinase K and RNase A. DNA from bound (IP) and unbound fraction (input) was purified by Phenol-Chloroform-Isoamyl alcohol (PCI- 25:24:1) extraction followed by ethanol precipitation. qPCR was performed as mentioned above and % input calculation was employed to assess the relative enrichment.

#### SDS-PAGE and western blot

Cells corresponding to 2 units of OD_600_ were subject to protein extraction by TCA method. To the cell pellet 200 µl of 20% TCA was added and lysed by vortex at high speed for 5 min. The lysate was then collected onto a fresh tube and the glass beads were washed twice with 150 µl of 5% TCA. The washed elutes were added to the previous lysate and spun at 13000 rpm for 10 min. To the pellet 200 µl of Laemmli buffer was added and pH was adjusted using 2 M Tris.

The dissolved protein pellet was then denatured by boiling at 95°C for 5 min. SDS-PAGE was performed followed by a semi-dry method of transfer (Power Blotter system/Invitrogen) to PVDF membrane for western blotting. The blots were then probed with primary TAP-tag antibody (Genescript A01435, 1:5000) and HRP conjugated secondary anti-rabbit (Abcam ab97051, 1:10,000). For probing Rtt103 × 13myc, rabbit Myc (Abcam Ab9106 1:1000) primary was used. The signal was detected by ECL reagent (BioRad) and imaged in ChemiDoc Imaging system by BioRad.

#### Northern hybridization by slot blotting and southern

For RNA slot blots, 10 μg of denatured RNA was loaded onto Hybond N+ membranes (Amersham) using slot blot manifold Hoefer PR648 (Amersham). For southern blot, 3 μg of over-night digested XhoI genomic DNA was loaded. After transfer, blots were auto cross-linked via Stratagene UV Stratalinker 1800. The probes and whole hybridization procedure were carried out according to the manufacturers instruction for DIG-High prime DNA labelling and detection starter kit I (Roche 11,745,832,910). For Tel probe, terminally digoxigenin labelled d(GT)_30_ was used.

## Supplementary Material

Supplemental Material
